# The FAIR AOP roadmap for 2025: Advancing findability, accessibility, interoperability, and re-usability of adverse outcome pathways

**DOI:** 10.1016/j.comtox.2025.100368

**Published:** 2025-09-01

**Authors:** Holly M. Mortensen, Maciej Gromelski, Ginnie Hench, Marvin Martens, Clemens Wittwehr, Saurav Kumar, Vikas Kumar, Karine Audouze, Vassilis Virvilis, Penny Nymark, Michelle Angrish, Iseult Lynch, Stephen Edwards, Barbara Magagna, Marcin W. Wojewodzic, The FAIR AOP Cluster Working Group

**Affiliations:** a Public Health and Integrated Toxicology Division, Center for Public Health and Environmental Assessment, Office of Research and Development, US Environmental Protection Agency, Research Triangle Park, NC, USA; b QSAR Lab Ltd. ul. Trzy Lipy 3, Budynek B, 80-172 Gdansk, PL, Poland; c RTI International 3040 E Cornwallis Rd, RTP, NC, USA; d Department of Bioinformatics - BiGCaT, NUTRIM, Maastricht University, Maastricht, NL, the Netherlands; e European Commission, Joint Research Centre (JRC), Ispra, IT, Italy; f IISPV, Universitat Rovira I Virgili, Tarragona, ES, German Federal Institute for Risk Assessment (BfR), Berlin, DE, Germany; g Université Paris Cité, HealthFex, Inserm, FR, France; h Biovista, Charlottesville, VA, USA; i Institute of Environmental Medicine, Karolinska Institute, Stockholm, SE, Sweden; j Center for Public Health and Environmental Assessment, Chemical Pollutant Assessment Division, US Environmental Protection Agency, Durham, NC, USA; k Environmental Nanosciences, School of Geography, Earth and Environmental Sciences, University of Birmingham, UK; l Center for Computational Toxicology and Exposure, Scientific Computing and Data Curation Division, US Environmental Protection Agency, Durham, NC, USA; m GO FAIR Foundation, Rijnsburgerweg 10, 2333 AA Leiden, NL, the Netherlands; n Cancer Registry of Norway & Department of Chemical Toxicology, Norwegian Institute of Public Health, Oslo, NO, Norway

**Keywords:** AOP, Annotation, Artificial intelligence (AI), Biomedical, Computational toxicology, Data domain, Data integration, Database, Environmental health science, FAIR, Gene, Protein, Proteomic, Mechanistic, Natural language processing, Next generation risk assessment, New Approach Methods (NAMs), OMICs, Pathway, Toxicology

## Abstract

Adverse Outcome Pathways (AOPs) describe the mechanistic interactions of biological entities with a stressor (chemical, nanomaterial, radiation, virus, etc.) that produce an adverse response. How these interactions and associations are catalogued contributes to our ability to understand mechanistic effects and apply this knowledge to New Approach Methods (NAMs) that have the potential to reduce animal testing in chemical, biological, and material safety assessments. Making AOP data align with FAIR (Findable, Accessible, Interoperable, and Reusable) metadata standards relies on technical tools that implement and process AOP data and related metadata, and the establishment of coordinated and consensus computational bioinformatic methods. Herein current efforts in addressing the FAIRification of AOP mechanistic data and metadata, as well as the international, collaborative efforts to document, and improve the (re)-use and reliability of AOP information will be described. These coordinated efforts contribute to the establishment of a directive for the processing and storing of standardized AOP mechanistic data in the AOP-Wiki repository, and application of these data to next generation risk assessment.

## 1. Introduction

The Adverse Outcome Pathway (AOP) framework (Ankley, Bennett et al., 2010 [40] Villeneuve, Crump et al., 2014 [41]) has been an important concept in toxicology in that it has improved our ability to consistently organize, describe and communicate mechanistic and biological events that lead to human and ecological adverse health outcomes. The ability to be precise and consistent in our reporting standards across areas of biological observation has motivated efforts to align AOPs with the FAIR principles (Findability, Accessibility, Interoperability, and Reusability, ***see*** Box 1) [35]. The rationale for doing so varies across user groups, where AOP creators, users, and tool developers may have different requirements and research objectives.

In a preliminary look at the application of FAIR principals to AOPs, Wittwehr et al. [36] articulates the imperative that AOPs be deeply rooted in the FAIR principles, and posit that the AOP framework, which is central to linking measurable toxicological mechanisms to health outcomes, must advance to become fully *machine-actionable* in order to achieve its full potential in regulatory toxicology. The AOP-Wiki is the “primary repository for qualitative information for the international AOP development effort” (https://aopwiki.org/). The AOP-Wiki has been identified as needing FAIRification to enhance its visibility and improve trust among a diverse array of stakeholders (e.g. risk assessors, risk managers, policymakers, and the scientific community). This need for modification and improvement of AOP standards within the AOP-Wiki repository stems from the pragmatic motivation for improving AOP expert observational information derived from the crowd sourcing exercise to include mechanistic, biological information necessary for predictive toxicology at the level of granularity needed to enable New Approach Methodologies. The representation of complex information within the AOP-Wiki as “measurable steps… from a molecular perturbation to an adverse outcome for an organism or population” (https://forum.aopwiki.org), presents opportunities to apply FAIR standards to AOPs at various levels. Trust in the AOP-Wiki, however, and the tools that implement AOP-Wiki data, is dependent on the credibility and quality of the information contained in the AOP-Wiki. Wittwehr et al. [36] identify several challenges that hinder the AOP framework’s impact, such as the lack of incentives for AOP developers and concerns about the credibility of AOPs. It is suggested that embracing FAIR principles can address these challenges by facilitating a structured exchange of information that enables more efficient decision-making processes. We believe that addressing the needs related to complexity and transparency for the purposes of data provenance and machine-actionability, *i.e*. the FAIRness of AOP information, can improve the credibility of those data and related metadata (from here on referred to as (meta)data. Here we define *meta*-data as differing from primary data in that the *meta*-data item provides *context* on how the primary data were generated or obtained, as well as providing the user (re-user) better *understanding* of the data.

The life cycle of AOP development can be described as a progression from “pragmatic simplicity” to “necessary complexity” to ultimately “informed simplicity,” (Knapen, 2021). This concept describes the maturity of an AOP from a straightforward representation that may lack detailed context, to fully considered complexity of biological and toxicological data, and finally to an easily understood and scientifically robust AOP (Knapen, 2021, [36]). One goal to facilitate this process is the documentation of AOPs in a user-friendly and standardized format, without sacrificing the reporting of critical scientific information. Wittwehr et al. [36] discuss the need for inclusion of data provenance information, and underscore how data transparency and context preservation of underlying source data contributed in the AOP development process. It is imperative for users of the AOP-Wiki to have a comprehensive understanding of the origins of AOP evidence (e.g. data content), normalization, and context in which the information was assessed for any given AOP. This includes documenting the methodology employed (e.g. search strategies; data sources used), and scope or problem formulation under which the AOP was assembled. Providing such provenance information ensures that AOP consumers can assess the reliability and relevance of AOPs, and the quality of the underlying data for their specific needs. Further, requiring that the AOP data is FAIR further ensures that the (meta)data supporting an AOP are FAIR throughout the data lifecycle. The need for transparency and machine actionability supports better tool development with information obtained from the AOP-Wiki. FAIR concepts and criteria and standards, like those outlined in Box 1 and Table 1, are important for both the data going into the AOP-Wiki repository and for the processing of primary AOP data outside of the AOP-Wiki, in establishing the trustability in AOP information and its use in regulatory toxicology.

Wittwehr, et al. [36] calls for a balance between the simplicity needed for practical use and the complexity required for scientific accuracy, advocating for a FAIR approach to AOP development that supports informed decision-making in toxicology. Some FAIR “quick wins” have been discussed (https://forum.aopwiki.org/) and could include, for example, linking researcher ORCIDs for AOP developers and coaches, including DOIs for referenced works, broadening the use of key event (KE) components, standardizing and harmonizing KE terms, and providing annotations of genes and proteins with commonly accepted identifiers or vocabulary (e.g. HUGO (HGNC), NCBI Entrez, or UniProt ID). Some of these items are frequently addressed by AOP authors and tool developers, but no clear standard or directive has been given for how increased coordination can be accomplished. Recently, several projects have been initiated to address aspects related to how AOP mechanistic content can be improved. It is the object of the FAIR AOP Cluster to be informed of the AOP scientific progress related to improving AOP data standards and the creative development of AOP tools. We are obligated to improve the mechanistic content of AOP information and thereby improve the AOP-Wiki repository health for the good of the AOP community.

The present paper will build upon concepts and emerging needs to ensure AOPs are theoretically sound and practically applicable in a digital and data-driven future. The outstanding issues in the development of a FAIR AOP knowledgebase identified by Wittwehr, et al. [36], are improved visibility, (re)usability and trustworthiness, whereby obstacles to implementation of FAIR principles are both the complexity of information and the multidisciplinary of stakeholders. It is the specific aim of the FAIR AOP Cluster Workgroup to improve FAIRness at the level of the mechanistic content of AOP (meta)data, hereby advancing AOPs from an information content perspective, and specifically in relation to the mapping of biological mechanisms. The exploration of how FAIR concepts relate and can be applied specifically to AOP information is one area of the FAIR AOP Cluster inquiry. Box 1 describes FAIR concepts that are relevant to AOP mechanistic data and FAIR AOP tool development. These concepts are not specific to AOP data, but general to the improvement and interpretation of data standards for AOP (meta)data and tools that implement these data and facilitate data manipulation. Several AOP tools have been developed to improve the interpretation, accessibility, structure and format of AOP information derived from the AOP-Wiki. Here we present existing AOP tools to highlight how the (meta)data are currently represented across these resources (AOP-Wiki Third party tools: https://aopwiki.org/info_pages/8), and provide an overview of existing approaches that address AOP biological or mechanistic-level complexity in order to establish areas in need of coordination and to reduce duplicative, community efforts associated with the structuring, processing, and modification of AOP information obtained from the AOP-Wiki. The AOP community and stakeholder groups posit varied approaches to improve the FAIRness of AOP mechanistic data, encompassing the importance of data format, machine-actionability and the need for coordination at the level of bio-entity mapping for FAIR validation, as well as specific needs for the maintenance of human interpretability. Herein, these FAIR AOP coordination efforts highlight the value in mechanistic AOP data and propose specific, recommended outcomes. Lastly, it is crucial this FAIRification process can be refined by recommendations outlined by the AOP community and stakeholder groups. The combined outcomes of these exercises will be in establishing a “roadmap” of essential items, in light of the state of the science at the current time, and future needs to improve AOP mechanistic information for use and acceptance by scientists and regulators.

## 2. AOP development addresses biological mechanism

Biological pathway construction, which shares many similarities and differences with the accepted AOP development process, may help to elucidate areas of focus for the improvement and clarity of FAIR standards for AOPs. AOP development, despite the implemented OECD standards and process as outlined in the AOP Developers’ Handbook version 2.7 [33], (https://aopwiki.org/handbooks), is varied given the nature of the information available and the objectives of the contributor. This varied AOP content is not unlike biological pathway information, which has been described to differ in terms of data-driven (DD) or knowledge-driven (KD) objectives. Traditionally, biological pathway construction has been described as “the process of identifying and integrating entities, interactions, and associated annotations, and populating a knowledgebase”, where DD pathway construction is used to identify gene and protein relationships using experimental data, and KD pathway construction involves the development of detailed knowledge in specific domains such as cell type, disease or system [34]. The AOP concept does not make this distinction, making an AOP more abstract in many contexts, but instead underlines that an AOP describes events that specifically “culminate in an adverse outcome (AO) considered relevant to risk assessment or regulatory decision-making” [33]. AOPs “do not describe every detail of the biology but instead focus on describing critical steps or check-points along the path to adversity, which are both *measurable* and have *potential predictive value* for regulatory application” [33]. Further, because AOP authors represent various stakeholder groups, including government, academia, and industry, who submit AOP information to the AOP-Wiki repository via the free text interface, the resulting collection of AOPs is highly variable and heterogenous in terms of biological scope, data content, structure, and level of annotation.

### 2.1. AOP submission and validation process

Currently, the AOP-Wiki repository facilitates interdisciplinary, public exchange of information related to adverse outcome pathways, and contains 521 AOPs (as of the writing of this article, April, 23 2025). Authors of 137 (26 %) of these AOPs seek recognition under the OECD AOP work plan, while 384 (roughly three quarters) are managed independently by parties other than the OECD, especially the Society for the Advancement of AOPs (SAAOP). However, both “types” of AOPs share KEs, Key Event Relationships (KERs), and ontology terms, which are important for successful AOP network identification. Among the AOPs on the OECD workplan, four levels of completion according to OECD exist (e.g. *1. Under Development; 2. Under Review; 3. Working Party on Hazard Assessment (WPHA)/Working Party of the National Coordinators of the Test Guidelines Programme (WNT) Endorsed; and, 4. ESCA Approved*). 105 putative AOPs (included the current 384 mentioned above) are not under active development but were originally established to enhance the utility of US EPA Toxcast high throughput screening data, and are currently “open for adoption”. The most recent version of the AOP-Wiki, version 2.7, was released on March 30, 2024. Amongst OECD-classified AOPs, 83 are Under Development, 20 are Under Review, and 35 WPHA/WNT Endorsed (https://aopwiki.org/metrics; *Note that because the group was only established in 2024, no AOP has yet been approved by ESCA, and the process for AOP approval by the group is yet to be defined). AOP-Wiki quality control, or “gardening” efforts, aim to manually identify and correct overlapping or repetitive AOP information across submissions. The presence of overlapping and repetitive information within the AOP-Wiki can affect the downstream utility, as well as the AOP author’s ability to determine the correct level of detail and scope of a given AOP.

Currently, the OECD has not taken a position on FAIR in relation to AOPs, FAIRness as part of their scientific review process, or specifically how mechanistic insights might be improved through the process of AOP FAIRification. Because the FAIRness of AOPs is currently not implemented or established through the AOP-Wiki submission process as a primary requirement for AOP developers, or secondarily through OECD review, the onus falls on a tertiary review to establish the level of FAIR in relation to AOP (meta)data, which is generally when the AOP is used directly or subsumed by an AOP tool. The effort to establish FAIR AOPs directly contributes to the speed at which AOPs can be developed and reused in new AOPs (Svingen, Villeneuve et al., 2021) [42], [], and subsequently, to the to the level of acceptance and trustability of AOP tools using data from the AOP-Wiki repository, and their acceptance and use by AOP users and stakeholder groups.

### 2.2. Stages of FAIR validation

There are several relevant FAIR AOP efforts at various stages of development and maturity. Much of this work is shared with larger FAIR initiatives, whereby the AOP information is one type of contributing data stream. Inspiration can be taken, for example, from the five “FAIR levels” described in [15] *1. FAIR Start; 2. FAIR Play; 3. FAIR Go; 4. FAIR Share; and 5* – *FAIRest of Them All*. **FAIR Start** is expected for all datasets and addresses findability and accessibility. **FAIR Play** sets the groundwork for interoperability by providing links to resources that offer additional information about the lineage and methodology used to produce the data, and is presented in human-readable formats. **FAIR Go** incorporates the elements of the previous stages, but makes explicit references to the models that standardize data encoding, where datasets employ standardized terms and concepts. Importantly, here models and terminology may not, however, be available in machine-readable formats, suggesting that interoperability and reusability are facilitated through human mediation. **FAIR Share** moves to utilization of data models that are machine-actionable, in addition to incorporating the components listed in the preceding levels. Terms used to denote data elements are standardized, but may contain information that is not universally recognized or adopted by the relevant domain communities. **FAIRest of Them All** – represents the zenith of FAIRness within the European Commission’s suggestion, where datasets employ data models that are not only machine-readable, but also endorsed and maintained by the appropriate scientific domain community. Terms and concepts used for data encoding are machine-accessible and endorsed by the domain community, and interoperability and reuse by machine agents is maximized. This type of FAIR classification scheme is not uncommon for FAIR data efforts, and underlines our need to bin appropriately for certain usages, specifically related to machine actionability.

### 2.3. AOP tools can promote FAIR data format

The main repository of AOP information, the AOP-Wiki, provides the AOP XML, which is FAIR data format for AOP distribution. However, because AOP (meta)data requires further processing to interpret biological context and incorporate additional mechanistic content, AOP tools have been created around the consumption and processing of AOP (meta)data and the mapping of these data to other publicly available resources. Each AOP tool processes AOP data independently, and the choices made by each tool developer are unique. Fig. 2 illustrates the overlapping nature of information implemented by the existing AOP tools. It follows that the post-processed, AOP data available from each individual AOP tool is also unique to the tool, whereby versioning, processing steps, additional data sources, data formats, etc. are extremely relevant to the consistency and trustability of the resulting AOP information, as well as any potential for comparability across tools.

The FAIR AOP Cluster Workgroup recognizes the importance of data format to the FAIR AOP effort, where machine actionability is prerequisite. The coordination of FAIR, post-processed mechanistic content that should be directed back to the AOP-Wiki repository or other shared environment is an essential topic for this roadmap effort, and will assist AOP data in its approach to improved FAIRness. Fig. 3 illustrates the AOP tools discussed here, in terms of the FAIR Hourglass concept presented by Schultes (2023) [44] as it relates specifically to biomedical entity mapping to AOPs. For this specific data domain, the AOP tools overlap in content (as indicated in Fig. 2) but are approaching *FAIR Orchestration* with data integration, data sharing and automated services. Our ability to *F*ind, *A*ccess, make *I*nteroperable and *R*eusable all depend heavily on FERs, such as common data formats, identifiers and agreed upon data standards for access and reuse. One common theme for every FAIR effort is the establishment of a uniform “language” (e.g. format; template; nomenclature) that can be accessed and used by stakeholders. The FAIR principles [35] aim to make data both machine and human readable, but in which situations machine versus human readability is most appropriate for AOPs is dependent on the user’s needs at any given time and has implications to our interpretation of data and mechanistic content. For AOP information, a coordinated data format across AOP tools has implications to improved knowledge discovery, in the consistency and trustability of the resulting AOP information, and the coherency of AOP mechanistic and outcome interpretation. Further, the standardization of AOP (meta)data and tools can then be measured in the consumption of AOP information by risk assessment and regulatory activity. At present, the use of AOPs to support risk assessment and regulatory activities is still evolving and often complementary, rather than central to regulatory decisions. As of the writing of this manuscript, there are instances where AOPs have been used in hazard identification and Integrated testing and Assessment (IATA) [2,32]. Further, AOPs have informed risk and regulatory activities through the characterization and justification of mechanism for Endocrine Disruption (estrogen, androgen, thyroid, and steroidogenesis (EATS) pathways) [8], as well as informing screening, hazard assessments and prioritization efforts for Skin sensitization, Developmental Neurotoxicity (DNT) [7], Liver Steatosis and Pulmonary Fibrosis. Current AOP reporting is limited to voluntary documentation, and reporting via peer-review publication, with no agreed-upon and standardized reporting framework.

Human interpretability and machine readability are interrelated and both essential in the organization of AOP data, but can serve different purposes and meet the needs of different user communities. While data formats for machine readability, such as semantic web format [4] and ontology annotations [10], enable structured, computational processing of AOP information that can be interoperable with other (meta)data sources, and re-usable or reproducible, human interpretability focuses on increasing clarity to ensure that the information can be understood and used directly, making the data directly human-readable for researchers and decision makers that are not implementing computational approaches to understand the data. An illustrative example is that of AOP 392 (https://aopwiki.org/aops/392), which is somewhat of an intermediate example between human and machine readability, and describes a “Hub-AOP” for inflammation. The Hub-AOP can be thought of as a segment of a biology that is useful for integration and KE re-use, where AOP 392 re-uses previously described KEs (KE 1496, KE 1497). In addition, much of the development of AOP 392 has been focused on the re-usability through enriched annotation to external data sources (e.g. GeneCards the human gene database: https://www.genecards.org [31]), where links are embedded in the AOP-Wiki so that the user can directly explore the supporting biological information through point and click readability ([6,22]; *AOPs to model infection, inflammation and coagulation in COVID 19*; https://www.youtube.com/watch?v = D-nTwWIZRWM). However, in the AOP 392 example, within-text annotation with compact IDs does not directly improve machine readability unless there is development of FAIR AOP tools capable of interoperating with these annotations. However, these kinds of human-interpretable formats are seen as an improvement toward integration with machine-readable standards across AOPs that increase the accessibility and functionality of AOP data for a broader range of users. A dual approach in balancing human and machine readability serves to maximize the potential of AOPs in both scientific research and regulatory applications, thereby facilitating the interdisciplinary collaboration that continues to motivate the AOP framework and AOP-Wiki development (Carusi, Davies et al., 2018) [45]

#### 2.3.1. Machine-actionability of AOPs

Nevertheless, to ensure that information and data from the AOP-Wiki can be shared, integrated, and reused effectively, machine-actionable formats are essential, especially with regard to AOP tools built upon AOP information. Two areas of importance for progress toward machine-actionable AOPs are *1. the use of persistent identifiers and standardized nomenclature; and 2. the mapping of biological entities, which includes semantic mapping to ontologies*. The use of globally unique, persistent or compact, persistent identifiers and standardized nomenclature is critical to achieve machine readability. An identifier is first defined as a “single unambiguous string or label or name that references or identifies an entity or object (that can be a publication, a database, a protein, a gene, etc.)” (Laibe and Le Novere, 2007) [46]. According to Laibe and LeNovere (2007) [46] an object identifier must be: *Unique*: never assigned to more than one object; *Perennial*: constant and permanent; *Standards compliant*: must conform to existing standards (e.g. unambiguous URI); *Resolvable*: able to be transformed to online locations that store the object as data or metadata, and *Free to use*: free to create and use for everyone. In addition, the ideal identifier is “semantic-free”, where InChIs [19] could be one possible, though debatable, exception. Traditionally, URLs have not been thought to be usable, as they change over time. The Digital Object Identifier (DOI) has been used widely by the scientific community, though other systems have failed (e. g. BIOPAX, PURL) because of structural limitations that do not allow them to avoid one of the categories listed above. The use of compact identifiers for AOP (meta)data have been discussed recently at the INTOXICOM workshop [18], and a draft guidance document is under development https://github.com/egonw/compact-ids-in-reports. In the case of compact IDs, the URL is combined with an agreed upon community standard nomenclature to create a *resolved* service, or locally unique id (LUI). The URL then allows the service to *resolve* the compact ID into a link to the database and the LUI acts as an extension to redirect to the database to match the record.

#### 2.3.2. Biological entity mapping

Related, the mapping AOP (meta)data to external data sources and nomenclature systems enhances computational readability by defining clear relationships between entities (*i.e*. biological entities and events). This process improves how software and computational tools can effectively use, analyze and visualize AOPs. *Entity mapping*, also known as *normalization* or grounding, involves mapping text strings to a standardized, unique identifier within an ontology or database. By aggregating multiple mentions of the same concept under a single identifier, this process enables structured information extraction and facilitates knowledge discovery. Though performing manual entity linking on large information corpora is not feasible because it requires domain expertise to perform, automated approaches to entity linking can be broadly categorized into lexical matching and learning-based methods. A lexical matching approach relies on linguistic rules, patterns, and dictionaries to map concept mentions to their respective identifiers. Prominent lexical-based systems include CLARIT, SAPHIRE (Bhattacharya, 2024) [47], and MetaMap (Aronson and Lang, 2010) [48], for instance, are used to annotate concepts in PubMed, MedLine index using Medical Subject Heading (MeSH) identifiers (https://www.nlm.nih.gov/mesh/intro_indexing.html). While rule-based systems are efficient, they often fall short in capturing semantic meaning and struggle to link mentions that require contextual understanding. In contrast, learning-based approaches initially employ linear classifiers that are trained on Term frequency-inverse document frequency (TF-IDF) representations of mentions and their contexts. The use of models (e.g. CNNs, BiLSTMs, and Transformers) to convert raw data and text into numerical or vector representations has become more prevalent in the capture of semantic context with the advent of deep learning, and vector-based embeddings of context and mentions. These embeddings enable the use of binary or multiclass classification models to disambiguate similar lexical mentions based on contextual information.

#### 2.3.3. Use of ontologies to elucidate AOP mechanism and create FAIR AOPs

Ontologies provide standardized vocabularies for describing relationships between biological entities, enhancing data reusability and interoperability of AOPs across different tools/platforms. By incorporating ontologies, AOPs become more FAIR and machine-actionable, improving their mechanistic content and usability in regulatory decision-making. In ontology development, cross-referencing is done manually, as the terms require extensive review and validation by experts to assure consistency and accuracy. A well-developed ontology should also extend AOP mechanistic descriptions and metadata by linking KEs and key event components (KECs) to existing biomedical entities. This approach has been demonstrated through the mapping of KEs and KECs to ontologies [11,26], which enhance the mechanistic depth of AOP descriptions. While the AOP-Wiki does not explicitly display KE and KEC mapping to ontologies, these mappings are embedded in the AOP-Wiki XML and other AOP tools (e.g. AOP-DB; AOP-Wiki EXPLORER). Ives et al. [11] conducted the initial mapping of terms in AOPs, where KEs were assigned ontology terms. These KEs were represented as biological event components, comprising a biological object, process, and action, and were linked to corresponding ontologies. In contrast, AOP-DB integrates multiple resources containing AOP information using an entity-mapping approach and extends the ontology mapping to gene and protein ontologies [21,26]. This ontology-based structuring of AOPs has been further developed into RDF (Resource Description Framework) representations in a triple format AOP-Wiki RDF ([17]; https://bio.tools/aop-wiki_rdf) and the AOP-DB RDF [20]. Entity mapping is a critical step in structuring AOP information, enabling enhanced findability and interoperability at a highly granular level that allows automated validation of the fairness and completeness of AOPs using custom SPARQL queries. The AOP-WIKI EXPLORER [14] advances this process further by including detailed descriptions of AOPs and KEs, extracting entity mentions from unstructured text using deep learning methods, and normalizing these entities against relevant ontologies and databases. Extraction and normalization of key event terms improves the level of granularity at which information can be linked among AOPs, KEs, and external resources. Overall, the process of ontology mapping to AOP data and metadata improves computational interoperability and has the potential to improve mechanistic understanding through iterative data processing, which allows users the option of data layering, and visualization of different data types on top of the original, expert described AOP. Visualization tools further aid in data exploration of AOPs, and offering intuitive graphical representations that facilitate communication of intricate data and relationships within the AOP framework, contributing to a deeper level of trust among users [36].

#### 2.3.4. Relational and graph databases, semantic integration and usability concerns of AOP data

Relational databases have been used extensively to organize AOP (meta)data into tables with rows and columns, and are suitable for highly organized datasets with defined relationships between entities. However, relational databases can be of limited use when working with areas of abstract, highly connected or detailed AOP knowledge, where relationships between various biological events, stressors, and adverse outcomes can form complex networks. Though it is certainly possible to represent data from relational database structures as a network, graph databases implement the graph structures themselves to represent data as nodes (entities) and edges (relationships), which makes them ideal for capturing AOPs and the complexity inherent in AOP interactions. A widely-used framework for representing graph data is the RDF, which describes data in triples (subject, predicate, object). RDF is well-suited for AOPs because it enables the linking of data from diverse sources, ensuring interoperability. However, to be completely machine-actionable, RDF data must be semantically modeled using the RDF Schema (RDFS) and the Web Ontology Language (OWL). These standards allow for the definition of class, properties and relationships that describe the structure and meaning of data and knowledge, enabling more precise computational analyses. Another graph-based database model, the Labeled Property Graph (LPG), allows for increased flexibility with queries and the exploration of relationships between multiple layers or depths of processes and concepts associated with AOPs. Both RDF and LPG have specific costs and benefits when building AOP knowledge graphs. Recently, concern for the usability of these methods has indicated a continued need for less computationally intensive applications like graphical user interfaces for some user groups.

#### 2.3.5. FAIR implementation profiles in advancing AOP FAIRness

FAIR Implementation Profiles, or FIPs, are tailored frameworks that document and facilitate the implementation of FAIR principles in a specific domain of applicability, or *community of practice*. To this end, tools like the FIP Wizard and FAIR Connect [9,16], maintained by the GO FAIR Foundation (https://www.gofair.foundation/) offer user-friendly solutions for implementing FIPs effectively. In the context of AOPs, one can imagine a FIP as a guideline for AOP developers and contributors to ensure that the data, metadata, and other supporting resources adhere to FAIR principles. Moreover, a FIP can be created in three different ways: i) to specifically declare the current status of implementations (called the *AS-IS FIPs*); ii) to decide on the future use of FAIR Enabling Resources (called the *TO-BE FIPs*); or iii) to recommend the use of a minimal list of FAIR Enabling Resources for all members of a larger community (called the *Reference FIPs*, in short *RFIP*) (https://wiki.gofair.foundation). We believe these profiles can serve as a practical bridge between the theoretical FAIR guidelines and their operationalization within the AOP Framework.

FAIR Enabling Resources (FERs) can be understood as the building blocks of FIPs—the decisions and choices made for each specific FAIR principle. These may include services, tools, specifications, or data policies that are essential for operationalizing the FAIR Principles. Such resources provide critical functions to achieve levels of FAIRness and are explicitly tied to one or more FAIR principles, ensuring that data becomes actionable and reusable. For instance, a trivial example of an FER that is already in use for the F1 principle (***see*** Table 1) [35], which focuses on ‘globally unique, persistent, resolvable identifier services,’ is simply the AOP-Wiki Identifier (AOP ID). It serves as a unique identifier for AOPs, ensuring their findability and accessibility. On the other hand, a more advanced example of FER can be the AOP-KB itself, serving as a searchable, query’able service to retrieve specific data and metadata on AOPs (as implementation for the F4 principle), see Table 3

## 3. A ‘FAIR shift’ within the AOP development and endorsement process

The development and further endorsement of AOPs by the Organisation for Economic Co-operation and Development (OECD) currently follows a multi-phase process, beginning with knowledge assembly, followed by scientific review, and culminating in formal approval by the responsible OECD committee [24]*, Guidance Document for the Scientific Review of Adverse Outcome Pathways*]. Despite its comprehensiveness, this process has yet to explicitly incorporate FAIR validation, leaving a significant gap in aligning AOP development with modern scientific and technological standards. Ultimately, the overarching future goal should be a ‘**FAIR shift**’ that integrates a subprocess within AOP development, requiring compliance with FAIR principles from the initial knowledge assembly phase, and then supporting the review phase (Fig. 4).

Given that FAIRness is increasingly recognized as a fundamental requirement across all scientific disciplines, it is crucial that future AOP development aligns with these principles. Integrating FAIR principles into the AOP development and endorsement process can address critical challenges by beginning with the standardization and unification of tools and practices available to AOP developers for managing data and metadata. This includes ensuring accessibility, provenance, and findability, and ultimately overcoming barriers to machine-actionability through the integration of specific services and technologies that support automated processing. This shift towards FAIRness is not just a technical enhancement but a foundational step towards ensuring that AOPs remain robust, reusable, and effective in meeting scientific and regulatory needs (see Fig. 5).

### 3.1. Supporting AOP development through the AOP FIP

Incorporation of FIPs can significantly enhance the AOP development and endorsement process. In this context, the FAIR shift can utilize an idea of a general ‘AOP FIP” as a practical tool to embed FAIR principles throughout the AOP lifecycle. The AOP FIP, through providing specific FERs, would ease the process of AOP development by serving choices and solutions that guarantee adhering to the FAIR principles at the early stage of AOP development. The AOP FIP would facilitate machine-actionability by standardizing FERs on data and metadata formats and employing persistent identifiers and services, ensuring that AOPs are computationally accessible, enabling automated validation and integration through other tools. Furthermore, the FIP would streamline collaboration by providing a unified FAIR framework that aligns diverse choices of the AOP community, including authors, reviewers, and regulators. This would eventually reduce redundancy and accelerate development. Moreover, the evaluated solutions and choices documented in the AOP FIP can serve as foundational tools for other AOP components, such as KEs and Molecular Initiating Events (MIEs). These already established solutions can lay the groundwork for developing specialized FIPs tailored specifically to KEs and MIEs, fostering even further the consistency within the whole AOP framework.

Lastly, and most importantly, we believe that the use of an AOP FIP as a guiding agent, both during the AOP development and review phases, can serve as a robust quality assurance mechanism. This approach would ensure that AOPs align with established FAIR standards, improving their transparency, reliability, and utility before they proceed to scientific review.

A Reference AOP FIP has been initiated by the FAIR AOP Cluster, and in response to envisaged outcomes of the CIAO effort [6]. The term AOP description has been defined as a digital object type. This Reference Adverse Outcome Pathway FIP can be accessed using the FIP Wizard or as a machine readable nanopublication [16].

### 3.2. Future directions for AOP FIP integration

To maximize their impact, AOP FIPs must evolve to include automated verification systems to assess and validate the FAIRness of AOPs during the knowledge assembly and review phases. Regulatory alignment is equally critical, requiring collaboration with OECD committees to formalize FIPs as a criterion in the AOP endorsement process. Additionally, ongoing community engagement in continuous refinement of FIPs and maintaining their relevance to all new regulatory and scientific needs is crucial.

Embedding FIPs within the AOP development and endorsement process can ensure that the framework achieves a higher level of FAIRness, enhancing its credibility and usability. For regulators, this translates to more transparent and reliable data, facilitating informed decision-making. For developers, it means streamlined processes and easier integration of standardized resources, reducing redundancies and accelerating AOP creation. This integration is rather a critical step towards solidifying the role of AOPs as a cornerstone of modern toxicology.

#### 3.2.1. AOP community focused efforts on FAIRifying AOPs

##### AI4AOP:

Because the AOP development and assessment process can be onerous, and often prohibitive, in terms of time and resources required to document, develop and establish the necessary quality of information supporting an AOP, and related KE and KERs, the role of AI in AOP development is currently being explored. The AI4AOP effort was instigated [36] to explore all areas and domains, processes, and workflows in which AI can support, simplify, or speed up the ideation, creation, maintenance, promotion and quality checking of AOPs, specifically the five processes (1) AOP description, (2) KE description, (3) KER description, (4) overall AOP assessment, as well as (5) AOP review is essential for scaling the AOP framework to the extent necessary to be fully embraced by the toxicology community. In addition, AI4AOP is exploring the use of AI in relation to many other areas of improvement of AOP data and content structure, such as data integration and harmonization, peer-review, consensus building and expert and stakeholder identification and tracking, curation and resolution of KE and AOP networks, and systematic review and evidence mapping, for example. AI4AOP will of course identify existing initiatives that combine AI and AOPs, like e.g. the AOP-helpFinder (https://doi.org/10.1289/EHP4200, an AI-based tool to expedite the identification of links between environmental stressors and the biological events associated with AOPs) and ChatAOP (https://github.com/ideaconsult/ChatAOP, aiming to enable intuitive querying and generating summaries of toxicological knowledge to support decision-making and risk assessment).

##### AOP Ontology Development Group:

This group has been initiated to reexamine previous AOP ontology mapping efforts [11,26] and continue AOP ontology development (Adverse Outcome Pathway Ontology, https://github.com/DataSciBurgoon/aop-ontology), and works in collaboration with the Methods2AOP and FAIR AOP Cluster workgroups. This group will coordinate and improve the usage and mapping of AOP and related (meta)data to existing ontologies, as well as the creation of a standardized AOP ontology.

##### ELIXIR:

ELIXER is an organisation that unites life science resources from across Europe into a single, coordinated infrastructure. Its goal is to facilitate the management and sharing of all types of biological data, share expertise, and collaboratively develop best practices to ensure that data and information are easily accessible and interoperable for researchers across different disciplines. By connecting bioinformatics tools and resources, ELIXIR promotes open, reusable data that aligns with the FAIR principles. Through the Data and Interoperablity platforms, ELIXIR provides services which are also useful in the FAIRification of AOPs. For example, identifiers.org is one of the recommended interoperability resources as a provider of globally unique and persistent identifiers. The main components of the AOP-Wiki (AOP, KE, KER and Stressor) can be “resolved” using identifiers.org. Furthermore, FAIR Cookbook initiative provides a framework for developing guidance on FAIRification of data, tools or knowledge. The ELIXIR Toxicology Community has initiated FAIR Cookbook recipes associated with AOPs and the AOP-Wiki (https://elixir-europe.org/communities/toxicology).

##### EHLC AOP Standards Use Case:

The EHLC (Environmental Health Language Collaborative) was established through an NIH/NIEHS-EPA collaboration effort (https://www.niehs.nih.gov/research/programs/ehlc), with the vision to enhance the translation and knowledge discovery for environmental health sciences data. Through use case studies, the EHLC effort addresses community needs related to data harmonization, sharing, and interoperability, and in this way is helping to establish common data terminologies and standards. Current use cases focus on a broad level of scientific issues such as biomarkers and biological processes of exposure, and data discovery and harmonization (https://www.niehs.nih.gov/research/programs/ehlc/use-cases). Recently, an *EHLC AOP Standards* use case has been initiated and will address FAIR principles and consistency in AOP entity mapping and annotation for AOP tools. In June 2025, the EHLC Executive Committee will host experts in the overlapping areas of biomedical and AOP data usage and interoperability for a two-day interactive AOP standards workshop event. The focus of the workshop will be on the coordination, harmonization, and development of standardized competency and use assessment for AOP (meta)data with existing biomedical and knowledge information systems.

##### FAIR AOP Cluster:

The FAIR AOP Cluster was formed to address the need for FAIRification of AOP information. The FAIR AOP Cluster workgroup participants are an international group of government, academic and industry tool developers and model builders with a shared interest in the FAIR use of AOP information. For this reason, the recent focus of the group has been on the FAIRification of AOP mechanistic (meta)data, and FAIR contributing tools. Though the present manuscript describes how the workgroup interacts with other efforts to execute the mission to advance AOPs to become fully machine-actionable and achieve full usability in regulatory toxicology through the linking of measurable toxicological mechanisms to health outcomes, the FAIR AOP Cluster is tasked specifically in this reporting with the FAIRification of the AOP framework. It is the opinion of the workgroup that through the coordinated, improved FAIRification of AOP mechanistic data, the AOP framework, and subsequently AOP tools, will benefit by improved trustability and transparency.

##### Methods2AOP:

Methods2AOP focuses on changes to the AOP-Wiki data model to better represent test method information. The AOP-Wiki does not currently enable consistent description of test methods, method-related information or the linkage of KEs with test methods. Methods2AOP addresses this issue through the development of a process for the association of test methods with KEs. Assay methods (*in vitro* and *in vivo*) have been the focus of this mainly JRC, NIEHS, NIH, ECCC, and EPA collaborative work group. Linking relevant methods to specific KEs (and thereby implicitly to AOPs) will help build confidence in the use of NAM data for regulatory purposes. Methods2AOP aims to make the connections between test methods and KEs more explicit and visible. The Methods2AOP workgroup is working to identify a minimal information criterion and organize relevant methods inputs into an intuitive format [5,12].

##### OECD AOP Reporting Standards for Biomedical Data:

This OECD-WPHA project proposal, initiated by EPA and JRC, aims to coordinate the AOP FAIR reporting standards for biomedical data and annotation decided upon by the AOP community, establishing programmatic reporting of these (meta)data to the primary AOP-Wiki repository. This work will support the mechanistic underpinning of AOPs through improved data standards and the FAIR reporting of these data to support risk and regulatory activities.

##### Omics2AOP:

This project, initiated by the JRC and Tampere University’s FHAIVE, addresses the limited integration of omics data (like transcriptomics, proteomics, or metabolomics) within the AOP framework. The project aims to link genes, proteins, and metabolites to KEs and AOPs by leveraging established ontologies. Inspired by previous research demonstrating the use of Natural Language Processing and manual curation to connect gene sets to KEs, Omics2AOPs seeks to standardize omics data interpretation, support data integration efforts, and accelerate new assay development. This initiative has been proposed for submission to the OECD’s Working Party on Hazard Assessment, and actively engages the AOP community for feedback and collaboration.

##### SAAOP

The Society for the Advancement of Adverse Outcome Pathways (SAAOP) was originally formed to support the AOP-Wiki data and IT needs, and related issues to the storage and availability of the AOP-Wiki data. The SAAOP brings together the broader AOP community, and government stakeholders (OECD [25], JRC, US EPA, etc.). SAAOP held a series of webinars in 2022–2023, directed at the topic of Envisioning the Future of AOPs, which included specifically a focus on FAIRness of AOPs and related information. The 2nd SAAOP webinar (https://www.youtube.com/watch?v = 2BpkeTXUjEw) focused both human readability and machine readability for AOPs, with presentations from Drs. Anamaria Carusi, Interchange Research and Penny Nymark from Karolinska Institute, and standardization of ontologies and visualizations, presented by Dr. Visilis Virvilis from BioVista. The 3rd SAAOP webinar focused on the data model changes for the AOP-Wiki 3.0 presented by Dr. Jason O’Brien (https://www.youtube.com/watch?v = 2BpkeTXUjEw).

#### 3.2.2. Interpretation of AOPs using AOP tools and applications

Over the last few decades, with the decreased cost of generating biological and omics types of data and the rise of systems level thinking and bioinformatic interpretation, we have generated a vast amount of unstructured and scattered data, which have resulted in “siloed” and “difficult to access” information (Carusi, Davies et al. 2018). While some US-based projects like BioData Catalyst (https://biodatacatalyst.nhlbi.nih.gov/) and the NCATs Data Translator project (https://ncats.nih.gov/research/research-activities/translator) work to improve access to genome research data sets in service of biomedical research, these efforts have not addressed needs in toxicology, modern risk assessment or environmental health research areas. This approach is important if ONE HEALTH strategy should succeed (One Health). The lack of curation of toxicological and environmental health-relevant data sets is an additional challenge in the assessment of data quality. The AOP-KB has, since its inception, been designed to address these challenges, as both a repository and hub for software tools and applications that address the collection, formatting and evaluation of biological information related to AOPs. While the AOP-KB is the repository of the actual AOP knowledge, which can be accessed (in read-mode) by various third parties, the AOP-Wiki is the one (and so far the only) tool with which AOP-KB information can be added, edited and manipulated. From a software development perspective, the code base for the AOP-Wiki and AOP-KB are tightly coupled. A single Ruby on Rails application holds a schema and data models for the database that is referred to as the AOP-KB, which provides all data storage for the AOP-Wiki. Manual curation efforts are currently carried out by an AOP-Wiki gardener, who identifies areas of conceptual overlap between AOP author submissions; for example with conceptually similar KEs, the gardener determines when consolidation is possible, pending agreement between KE creators.

### 3.3. AOP-KB recognized tool repertoire

Collectively, all AOP tools have been developed to improve the interpretation, accessibility, structure and format of AOP information. The utility of AOP tools has been challenged by a diversity of audiences and stakeholders, e.g. AOP developer, informatician, tool developer, assessor, or regulator, etc. Further, a focus on improving the FAIRness of AOP information has expanded the approaches that make AOP data and metadata machine actionable [14,17,20,39] shifting the focus from individual tools or user groups, to that of improving AOP data format for interpretation, visualization, and query. In addition to the AOP-Wiki, there are currently seven, 3rd party AOP tools recognized by the AOP-KB. These are *AOPXplorer* (https://apps.cytoscape.org/apps/aopxplorer; https://github.com/DataSciBurgoon/aop_networks); AOP-helpFinder (Jornod, Jaylet et al., 2025 [49], Jaylet, Coustillet et al., 2023 [50], https://aop-helpfinder.u-paris-sciences.fr/); Kaptis (Lhasa) (https://wikikaptis.lhasacloud.org/#/aop); Biovista Vizit (https://aopkb.biovista.com/); AOP-DB and AOP-DB RDF [20,21]; AOP-Wiki RDF [17] and the AOP-networkFinder tool (https://aop-networkfinder.no/; https://doi.org/10.1093/bioadv/vbaf007). Of these seven, six are publicly accessible, and freely available, which we will highlight here. Kaptis (Lhasa) is a subscription product that provides data visualizations and (Kaptis) proprietary pathway information. A portion of the Kaptis data are openly accessible through the AOP-Wiki. Though this list is not all inclusive, it does include all of the secondary (or tertiary) tools that capture AOP data and are actively under development at this time. As a group, the AOP tools, described according to alphabetical order below, represent tools developed to improve the understanding, interpretation and FAIRness of AOP data relevant to mechanistic biological processes. Table 2 lists AOP tools that contribute to the mapping of AOP mechanistic processes. Fig. 2 illustrates the overlap of (meta) data associations and biomedical entities in each AOP tool listed in Table 2.

#### AOP-DB

The AOP-DB relational SQL database was an early effort by the US EPA to specifically map AOP KEs to gene and protein ontologies, and by extension to chemical, disease, species orthology, and assay information [26]. AOP-DB v.2 included all original data sources, with updated mapping of AOPs to tissue type, as well as population variability (SNP and “AOP haplotype”) information [21]. The US EPA AOP-DB searchable UI (aopdb.epa.gov-*deployed in November 2021, decommissioned in February 2025*) allowed users to access structured AOP-DB data and mappings to publicly available data sources (e.g. gene/protein, pathway, disease, chemical stressor, DTXID), providing various file formats (csv, json, xml) for download. Despite the EPA’s ongoing efforts to support NRC’s 2007 vision on Toxicity Testing in the 21st century (TT21C), and the utility that the AOP-DB UI provided with easy extraction and download of structured AOP information for the AOP community, AOP tool development continues to be challenged in communicating it’s utility to some audiences (pers. Comm. Vique Caro, Dir. US EPA/OSIM “*methodology to make gene-environment linkages is not mature enough for deployment and… approaches to using this data in risk management contexts is not yet well-understood*”). The entire 2021 version of the AOP-DB [21] continues to be available as a relational database structure from the US Office of Research and Development (https://catalog.data.gov/dataset/adverse-outcome-pathway-database-aop-db-version-2). Some of the AOP-DB tables have been semantically mapped [20] and the RDF structure is made available through the University of Maastricht (https://github.com/BiGCAT-UM/AOP-DB-RDF). Future implementation of the AOP-DB data will be in the agreed upon biological entity mapping that is the subject of this FAIR AOP Cluster workgroup effort, as well as EPA ToxCast assay to AOP mapping following the recommendations of the Methods2AOP effort. In addition, the EPA is currently providing OECD-approved AOP information (~75 AOPs) via the CompTox Chemistry Dashboard https://comptox.epa.gov/dashboard/.

*AOP-helpFinder v2* is a web service that implements an AI-based method to automatically screen the scientific literature stored in the PubMed database, thereby expediting the AOP development process (Carvaillo, Barouki et al., 2019 [52], Jornod, Jaylet et al., 2025 [49], Jaylet, Coustillet et al., 2023 [50]). AOP-helpFinder uses text-mining, graph theory and natural language processing (i.e., lemmatization and stemming processes (NLP) to identify and extract stressor-event (MIE, KE, AO) and event-event relationships. The method also provides confidence scores for each relationship, therefore supporting the weight of evidence. A new release is currently under development, where identified KER are crossed with other data sources, including AOP-Wiki, and interactive visualization networks are provided to the users. The tool is available at https://aop-helpfinder.u-paris-sciences.fr/.

#### AOP-KB

**Fig. 1.**
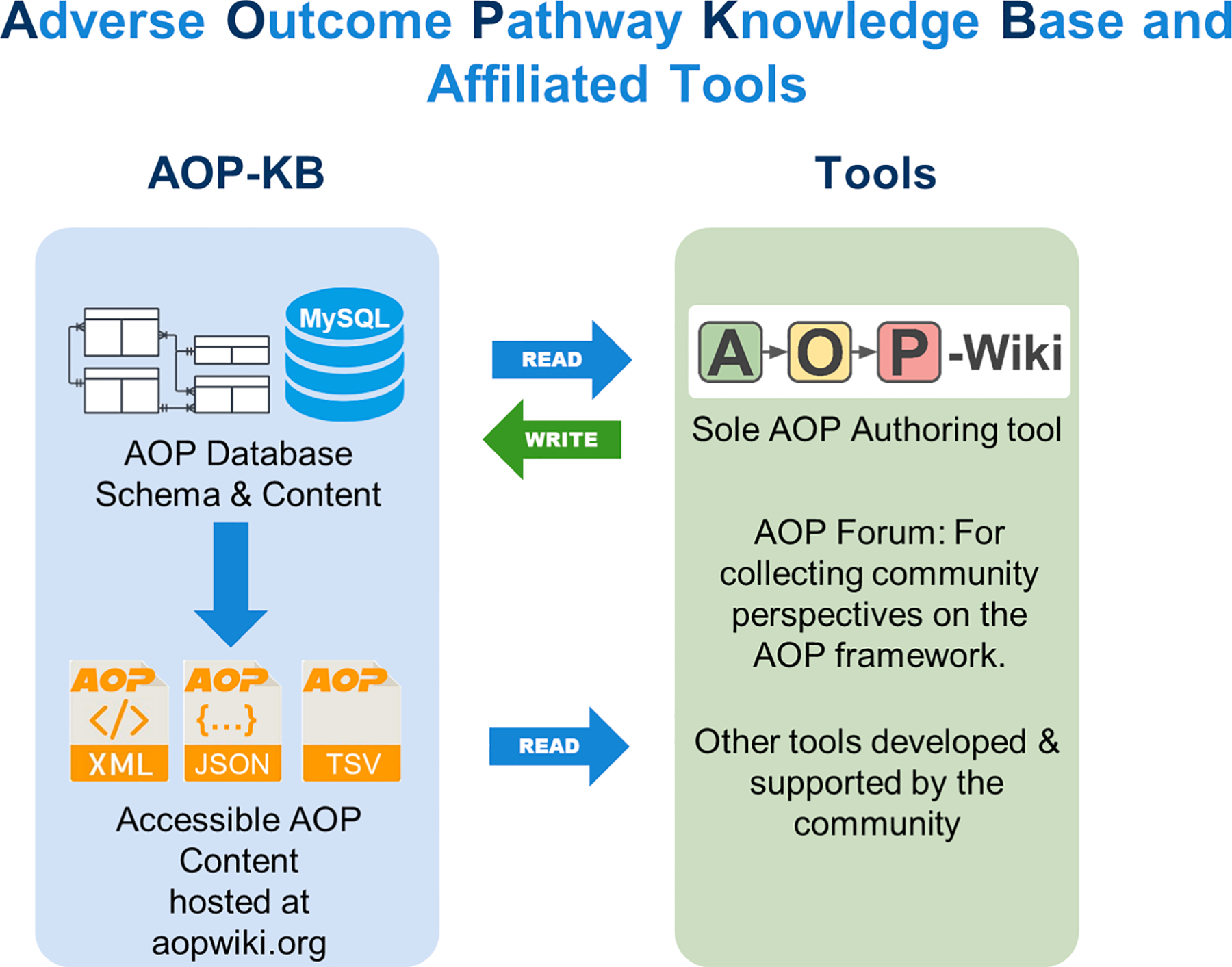
AOP-KB and affiliated AOP tools.

**Fig. 2.**
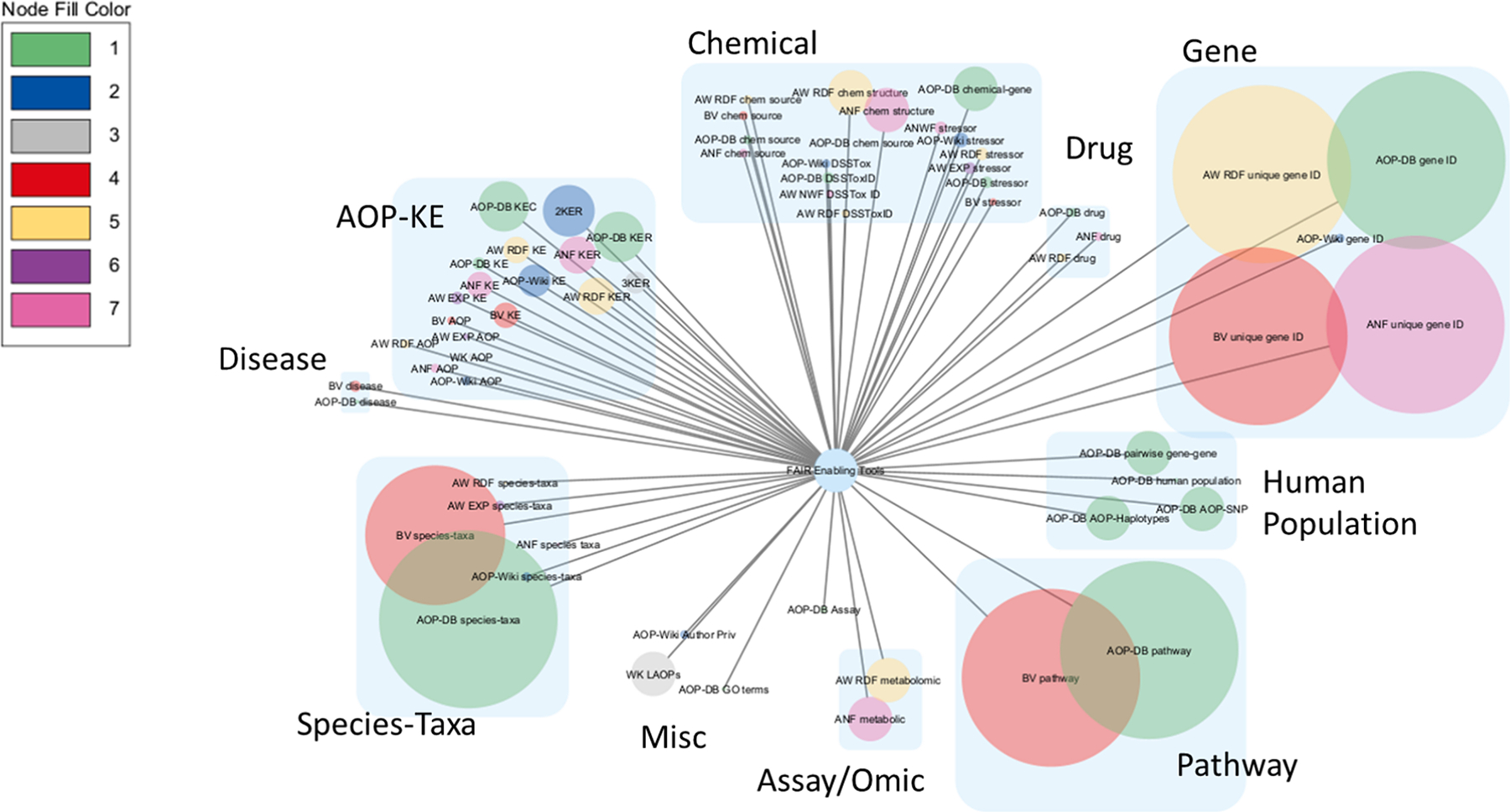
Network of data domains represented by each AOP tool.

**Fig. 3.**
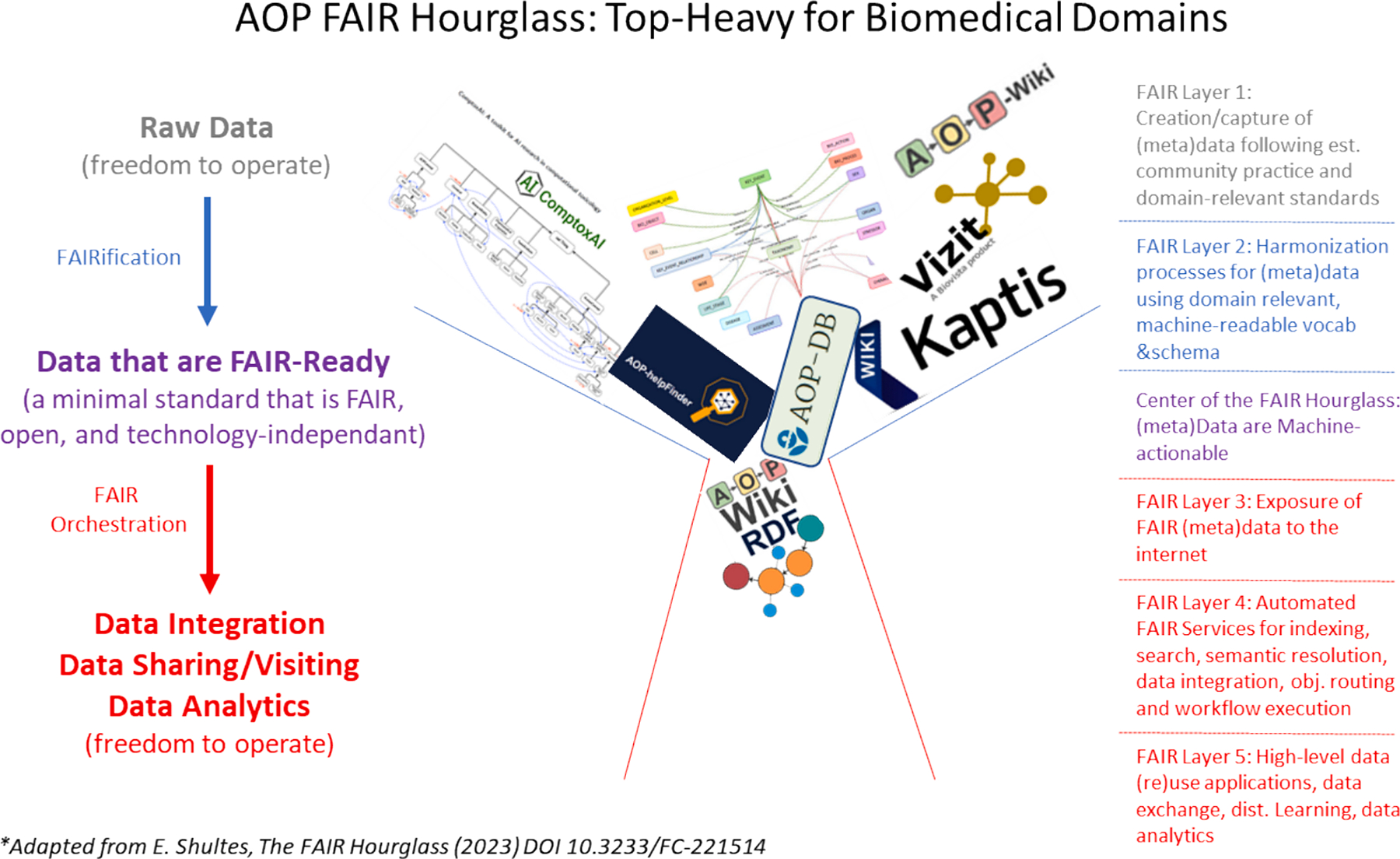
AOP FAIR hourglass is top-heavy for biomedical domains.

**Fig. 4.**
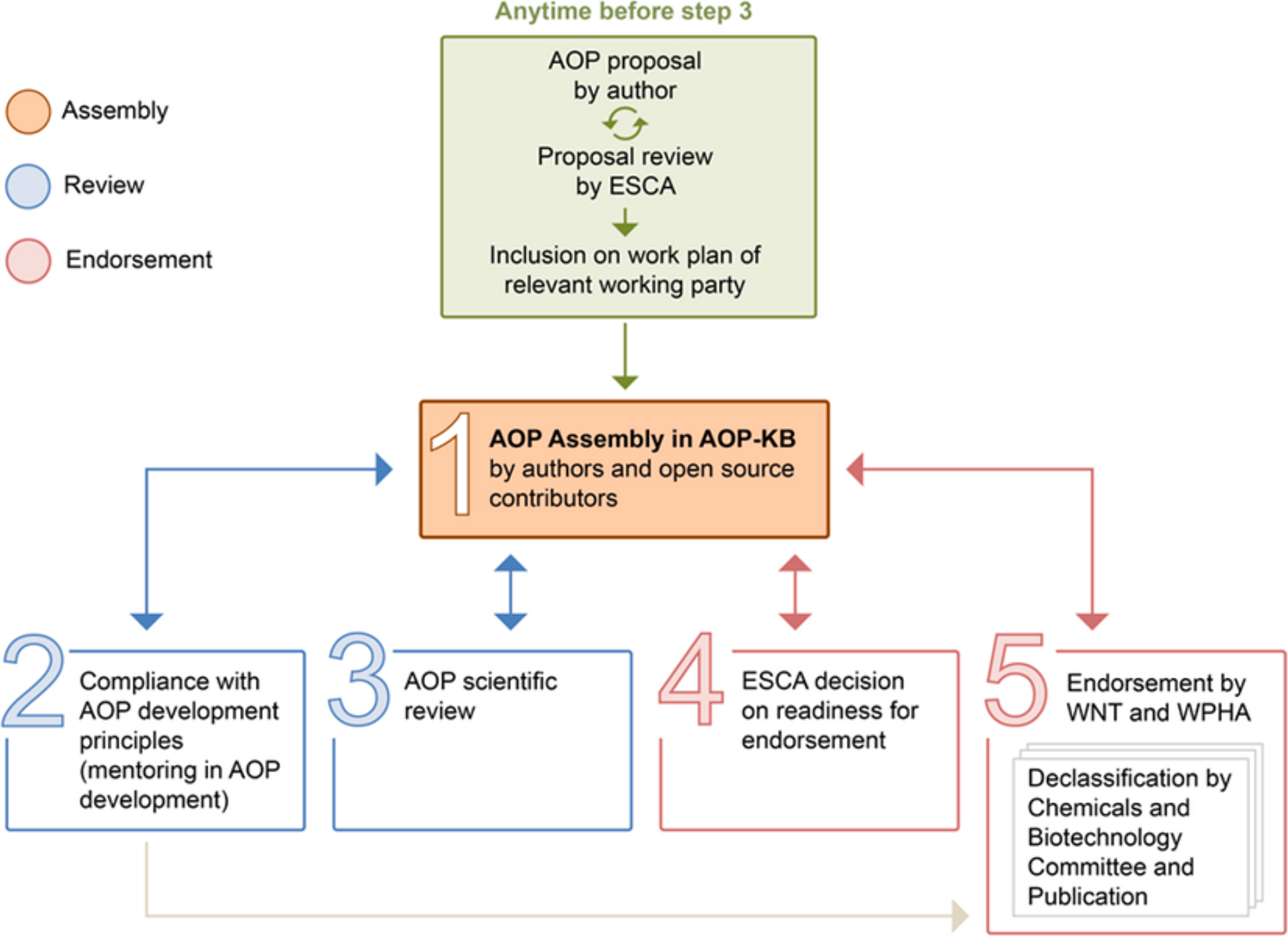
Schematic visualization of AOP development and endorsement process enhanced by the FAIR Shift towards compliance of AOPs with FAIR principles (adapted from https://www.oecd.org).

**Fig. 5.**
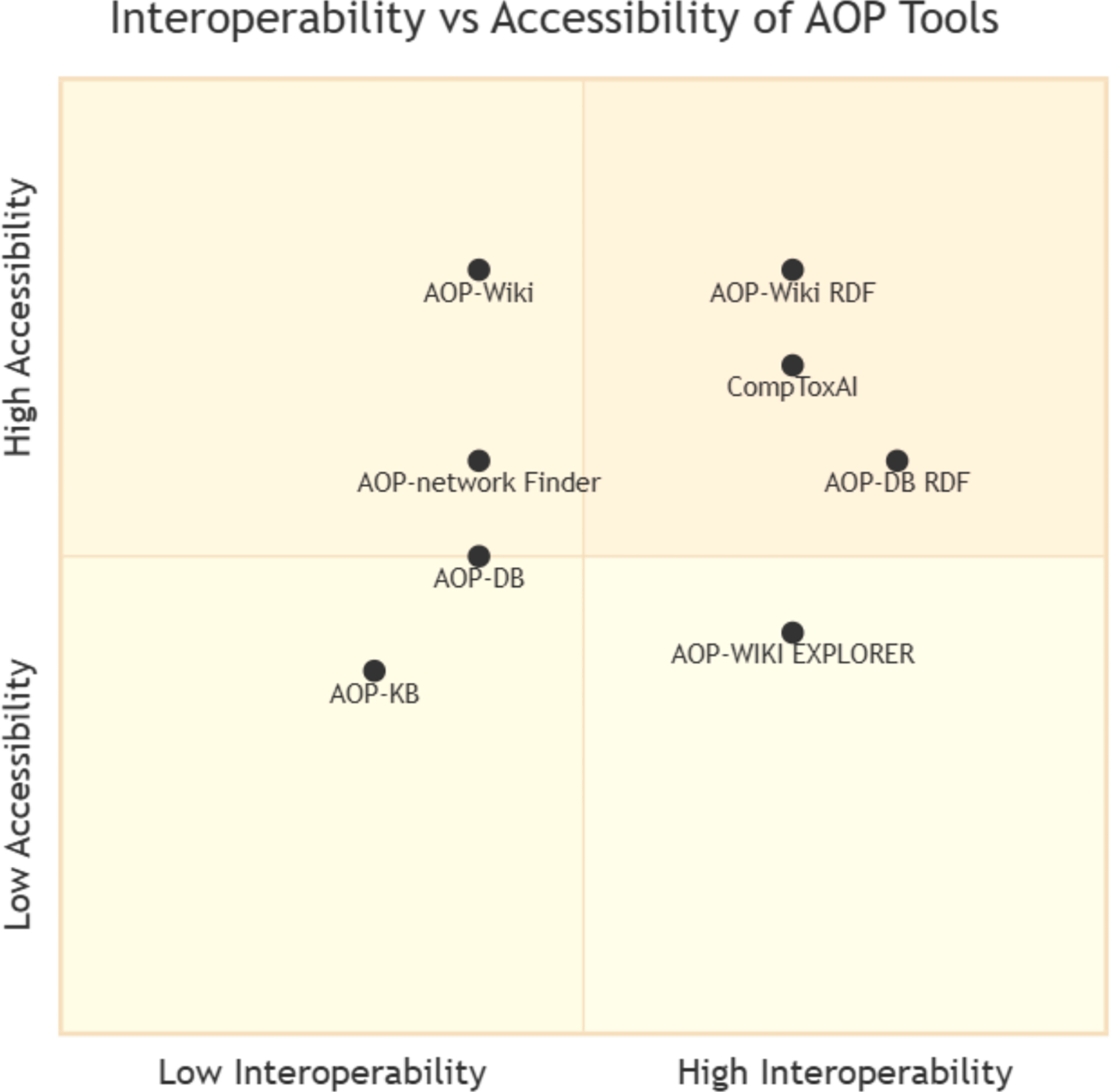
Qualitative assessment of AOP tools based on their interoperability and accessibility in the AOP FAIRification process.

The AOP Knowledge Base (AOP-KB, left side of Fig. 1, adapted from https://aopwiki.org/info_pages/10) is the primary central repository for all AOPs developed either as part of the OECD AOP Development Programme, or by the larger scientific community. The AOP-KB Tools (right side of Fig. 1) are a collection of web-based resources that are constantly undergoing development and refinement. Together, the AOP-KB and accompanying tools aim to bring together knowledge and evidence pertaining to how chemicals and other stressors (e.g. radiation, nanomaterials, viruses, …) induce adverse effects on humans and ecosystems. The AOP-Wiki serves as the primary and official authoring tool and user interface for submitting AOPs and their building blocks (KEs and KERs) to the AOP-KB. AOP contributions to the AOP-KB have been crowd sourced from the international AOP community, which represents many research and regulatory organizations, including government agencies and academic institutions. Financial support for ongoing maintenance and evolution of the AOP-KB and AOP-Wiki has come from the European Commission – DG Joint Research Centre (JRC), the US Environmental Protection Agency (EPA), Environment and Climate Change Canada, and the Organisation for Economic Cooperation and Development (OECD).

#### AOP-networkFinder v1

The AOP-networkFinder is a user-friendly web application designed by Norwegian Institute of Public Health to retrieve, modify, and visualize Adverse Outcome Pathways (AOPs) of interest (https://aop-networkfinder.no/;https://doi.org/10.1093/bioadv/vbaf007). It implements the AOP-Wiki RDF [17] programmatically via the AOP-Wiki Sparql endpoint provided through the AOP-Wiki service on the VHP4Safety platform (aopwiki.cloud.vhp4safety.nl/sparql/) to construct AOP networks by linking AOPs that share common KEs in a flexible yet controlled manner. Additionally, genes associated with these KEs are displayed to provide a more comprehensive view. The application allows users to export the constructed networks in various formats, including JPG, PNG, or directly to Cytoscape, enabling further fine-tuning and statistical analysis. This capability helps researchers efficiently explore relationships between KEs and visualize the overarching structure of an AOP, streamlining their understanding of complex interactions within pathways. A key feature of AOP-networkFinder is its ability to identify and suggest missing links between KEs, as well as display the closest neighbors (degree 1 and 2) of a given KE. This feature encourages the discovery of new relationships and the generation of novel hypotheses. The tool is particularly beneficial for researchers aiming to build extended networks while logging relevant information for repositories like Zenodo. Moreover, the AOP-networkFinder is inclusive in design, offering functionality that accommodates colorblind users. Its versatile export options and user-friendly interface make it an invaluable tool for comprehensively analyzing, visualizing, and enhancing AOPs, and was used successfully in the Partnership for the Assessment of Risks from Chemicals- PARC (adult neurotoxicity) and EFSA BrainHealth projects. V2 of the tool AOP-networkFinder with new functionalities are being developed. The tool undergoes further development to implement new features, such as filtration of Kes given applicability domain, connecting with ToxCast database and quantification of probability of activation AOPs given data using Bayesian statistics.

#### AOP-Wiki

The primary repository of AOP information, the AOP-Wiki (https://aopwiki.org/), integrates multiple ontologies from different domains and species with shared vocabulary as a common knowledgebase [11]. This process, of annotating AOP KEs with Process or Object (does not require both) and an Action was performed by Ives et al. [11] to promote visualization and analyses of genomic data in relation to AOPs, but also improves machine-readability. This approach was similar to that taken by the Monarch initiative to anchor phenotypic profiles by genes and diseases (Mungall, McMurry et al. 2017 [53]), through the use of ontologies. As described by Ives, et al. [11] this was performed in part to encourage the re-use of KE and KERs, thereby reducing redundancy. Importantly, while the annotation of KE and KEC has the potential to reduce redundancies between KEs in the AOP-Wiki, beyond the curation work provided by AOP-Wiki Gardeners, there is currently not an automated mechanism in place to directly reduce redundancies between KEs. FAIR improvements are ongoing to the AOP-Wiki., Release 2.6 included updates to creative commons licensing, which aligns with FAIR reuse standards and improved search features. Importantly, improved “findability” additions to the AOP-Wiki have preferentially focused on human-readable solutions (Hench et al., 2023 [54]), it has been suggested that the annotation of KEs with ontology or KOS terms (*i.e*. KECs) could improve the machine-readability of AOPs and have value for AOP network development efforts [21,26], (Wittwehr, Audouze et al. 2025 [55]). Additionally, the current version of the AOP-Wiki does not distinguish from data versus metadata, and allows users much flexibility in the capture and documentation of expert-derived, AOP relevant information. Though AOP-Wiki prototype work and plans have been initiated and relate to the update or alteration of structured information, no schedule has yet been determined. Plans for AOP-Wiki release 2.8 have been discussed, with a possible deployment of new EC features in 2026, but the timeline has not yet been confirmed. More generally updates to the AOP-Wiki are informed by the priorities and contributions of AOP interest groups, including SAAOP leadership, the SAAOP Knowledgebase Interest Group (Wittwehr, et al., 2025), and OECD ESCA. New feature selections are determined by the AOP-KB Coordinating Group (AOP-KB CG) based on needs expressed in AOP community spaces and available budgetary contributions.

*AOP-WIKI EXPLORER* is a labeled property graph (LPG) adaptation of AOP-Wiki. Where the LPG schema is adapted using neo4j to provide the user with cypher and natural language based queries to explore AOPs [14] which includes the ability to chain stepwise queries. Here, the AOP-WIKI EXPLORER extends the AOP-Wiki structure (e.g. AOP, key event, and Key event relationship) to include information like taxonomy, sex, biological organization level, assessment methodology, and others. The AOP-WIKI EXPLORER continues with the efforts to make AOP information machine-actionable, specifically with regard to graphical and node mapping, but in addition textual information processing using NER (Named Entity Recognition) to extract biological concepts such as gene, protein, chemical disease etc. and link KEs based on mentioned biological concepts.

*AOP-Wiki RDF* was created to systematically parse, filter, and use AOP-Wiki information, improve upon the existing Wiki structure while linking out to chemical and biological information [17], but also to specifically improve the machine-accessibility through semantic annotation that allows linkage through chemical or protein annotations to WikiPathways (Waagmeester, Kutmon et al. 2016 [56], Martens, Ammar et al. 2021 [57]). The SPARQL endpoint (https://aopwiki.rdf.bigcat-bioinformatics.org/sparql/) and SNORQL query UI https://aopwiki.rdf.bigcat-bioinformatics.org/ allow users the ability to access data in machine readable formats and perform chemical-centered queries.

*Biovista Vizit* was born out of the recent International Ciao collaboration [6,23,37], (https://www.ciao-covid.net/findings), as a Ciao initiative. Biovista Vizit uses over 16 different Knowledge Sources (Ontologies and Controlled Vocabularies) to generate a search space in the form of Biomedical term lists and present a search utility whereby users can generate network diagrams of their queries. In addition to the publicly available resources representing genes, disease, adverse events, drugs, lipids, MESH, compounds, and cell line, as well as Human Microbiome Project reference organisms, Biovista compiles additional controlled vocabularies and ontologies in-house.

*CompToxAI* is a toolkit designed to enable AI and data science research in computational toxicology. Through a collaborative agreement between the US EPA and the University of Pennsylvania, data from the EPA AOP-DB [21], DSSTox (Grulke, Williams et al. 2019 [58]) and several other data sources, have been integrated in this graph database tool, with core functionality described in Romano et al. [28]. The utility of CompToxAI was further refined in the analyses presented in Romano, Hao, et al. (2022) and illustrated in Mortensen et al. [21] where AOP data was used to infer individual and population level AOP SNP “Haplotypes” for liver cancer phenotypes in the absence of case-control data in human populations.

#### S^2^CIE:

Semantic, Syntactic and Context based Information Extraction is a real-time information extraction platform designed to allow users to prioritize literature and extract evidence using declarative grammar-based queries and contextual information of the subject matter. It utilizes the Open Domain Information Framework (ODIN) to pre-index terms defining both syntactic and semantic roles of terms within a database, enhancing search accuracy and efficiency. Before indexing, articles or abstracts are annotated with granular token-level information, including Part of Speech (POS) tagging, Named Entity Recognition (NER), dependency graphs, and similarity features mapping. This enhanced structured representation enables users to query information using customizable declarative rules allowing for more precise and context-aware extraction of relevant content. Additionally, articles and abstracts are embedded into numerical vectors, facilitating context-based searches beyond traditional keyword matching. By combining pre-indexing with horizontal scaling, S^2^CIE facilitates real-time searches across over 35 million PubMed abstracts. Unlike traditional keyword-based search queries, S^2^CIE enables users to define rules over surface tokens and dependency graphs to extract exact sentences and biological events with high precision. Its grammar-based approach makes it particularly effective for extracting biological evidence comprising complex biological relationships and mechanistic insights from scientific literature – critical for AOP development. (https://dev.s2cie.insilicohub.org/) (Kumar, 2025).

## 4. Discussion

One common area of overlap in all the AOP tools is the mapping of mechanistic processes to molecular identifiers (e.g. genes, proteins, transcription factors, biological pathway, disease, etc.), referred to here, collectively, as biomedical entity mapping. This annotation process is overwhelmingly accomplished by the mapping of KE to known ontologies and represents the “low hanging fruit” in our work of understanding the myriads of biological processes represented by AOPs. Though each AOP tool performs this process is a slightly different, unique way, each AOP tool begins the process from the primary iteration of expert generated AOP information derived from the AOP-Wiki XML, whereby some of these mappings are already present in the form of Gene Ontology (GO), Medical Subject Headings (MESH), and Chemical Entities (CHEBI), as described in Ives et al. [11]. A second and subsequent processing of the AOP-Wiki XML to ontology mappings extended to a relational database structure in the AOP-DB [21,26], and a Research Description Framework, or RDF [17,20], and then comparatively, to Labeled Property Graph, or LPG, implementing Natural Language processing and Large Language Models for cipher query to improve AOP search capabilities [14]. Recently, entity mapping has been extended to include annotation of gene sets for the purpose of further linking of toxicogenomic data to KEs [29]. Saarimaki additionally implements natural language processing (NLP) and manual curation to associate biological pathway, phenotype and GO terms in an initial matching and prioritization process for AOP-Wiki KEs.

The processes and data types implemented by each secondary mapping of AOP (meta)data by the AOP tool creators are indeed overlapping and currently un-coordinated, but indicate the importance of the mapping and annotation of biomedical entities to AOPs in general, and the need for continued and consistent re-use of AOP data in combination with biological data streams to inform mechanism. We recognize that the process of biological entity mapping should be prioritized and coordinated, in an effort to encourage the improved FAIR standards for AOP data, and in the preparation for novel and integrative data streams [3,14] and methods applications (e.g. The Human Cell Atlas [27]; Digital Human [38]; Virtual Human Platform for Safety Assessment [13]). The importance of a coordinated process at this secondary mapping level is again underlined in the continuation of what we refer here as tertiary (or quinary and beyond) mapping of AOP information. This level of AOP tool is then characterized as making use of the secondary mappings of AOP information. For example, AOP-Wiki RDF, AOP-DB RDF, and ComptoxAI are built on AOP-DB mappings, and the AOP-Network Finder subsequently on AOP-Wiki RDF mappings. It is clear that if this process were to continue unchecked, in addition to full or partial updates by any contributor, that the result would actually lead to disorganization and potential mistrust of AOP information at this level, not to mention repetition of effort, and derail the use of AOP information in 21st century processes to define NAMs, and risk and regulatory applications of AOPs.

The continued mapping of biomedical entities to AOP components and use by the community is currently limited because there is no existing workflow that stacks the curation process with domain expertise and tools. Furthermore, reliance on manual curation is a significant resource bottle neck and this limitation is increasingly recognized [1]. This underscores the need for a more community driven and scalable, automated approach that includes access to ontology management software (that allows modeling from multiple terminology resources, provenance tracking, and data migration between resources). The true challenge will be maintenance of any curation workflow within a trusted community of domain expertise and developers.

Moving from fragmented systems to a cohesive, community-driven infrastructure addressing the standardization of AOP (meta)data has led to global collaborative efforts to address AOP (meta)data in relation to biomedical entity mapping, including assay and methods and ontology mapping, as described above. These annotation efforts are pragmatic, and currently centered in the EU in connection with the EU-PARC (Partnership for the Assessment of Risks from Chemicals) Effort, which aims to develop next-generation chemical risk assessment to protect human health and the environment in support of the EU Chemical Strategy for sustainability and the EU Green Deal-Zero Pollution ambition.

PARC promotes computational approaches to both information curation and the FAIRification of AOPs. Tools such as S^2^CIE (Semantic & Syntactic Context-driven Information Extraction) and AOP-helpFinder (for literature prioritization) support data curation efforts. Tools like S^2^CIE enables the extraction of mechanistic information from biochemical literature, leveraging semantic, syntactic, and contextual cues to annotate literature at the token level with grammatical, entity-type, and syntactic graph information. To further support FAIR principles, AOP-sketchpad is being developed as a comprehensive, schema-agnostic platform for organizing AOP information, enabling rich ontology-based annotation. Additionally, efforts to develop a dedicated AOP ontology have been re-initiated, with a renewed focus on harmonised evidence representation.

In the US, the community-driven and participatory NIH-led Environmental Health Language Collaborative (EHLC) Effort was established to promote the use of common language, data standards, and data re-use in the environmental health sciences (EHS) and EHS-adjacent fields, in the spirit of the NIH – Data Management and Sharing Policy. EHLC’s recent AOP Standards Workshop aimed to raise awareness, enhance alignment across communities, and gather insights to guide future efforts—particularly in improving knowledge and data generation, and advancing the automated and semi-automated integration of data into AOPs. Outcomes of the 2-day event will be a call for proposals from participants working on emerging activities across biomedical and AOP domains. These community efforts will address how the AOP community can best leverage current scientific developments in the representation of biomedical resources and data usage to improve AOP annotation and mechanistic content, in concert with adherence to the general FAIR principles and the specific principles for FERs as outlined in Box 1 and Table 1.

The FAIR AOP Cluster Workgroup recently submitted a proposal to address AOP Reporting Standards for Biomedical Data that was supported by the OECD Advisory Group on Emerging Science in Chemical Assessment (ESCA) (OECD March 31st, 2025). How these standards will be developed and implemented by the AOP community to improve FAIR AOP (meta)data is the objective of these pragmatic efforts. These efforts will progress with input from the PARC, EHLC AOP Standards and FAIR AOP Cluster Working group in coordination with the SAAOP Coordination Group and in response to priorities set forth by the OECD Working Party of the National Coordinators of the Test Guidelines Programme (WNT) and the Working Party on Hazard Assessment (WPHA), and member countries. Through these collaborative efforts and with the help of the support of OECD, this work directly advances how AOP reporting and by association the tools used to collectively process primary AOP-Wiki data. Though, at this time, a completed pipeline that is agreed upon by the AOP community is beyond the scope of the current manuscript, future work will be in the coordination of a curation protocol for the mapping of mechanistic content to AOPs that includes biomedical entity annotation as a primary FAIR AOP resource, and the repository health of future iterations of the AOP-Wiki.

## Figures and Tables

**Table 1:** FAIR Principle indexing for each FAIR Enabling Resource (FER) Type, with corresponding definitions from the FIP ontology (https://w3id.org/fair/fip) (FER Type version 2.0.0, created by Barbara Magagna and Erik Schultes; GO FAIR Foundation (Dec 2023) [16,30].

FAIR principle	FAIR Enabling Resource Types	Definition
**F1**	*Identifier Service*	A **service** that provides for any digital object (1) algorithms guaranteeing global uniqueness, (2) policy document that guarantees persistent and (3) resolution of the identifier to machine-actionable metadata describing the object and its location.
**F2**	*Metadata Schema*	A **specification** that specifies the structured representation of metadata describing attributes of data or other digital objects in terms of semantics, syntax and optionality.
**F3**	*Metadata-Data Linking Schema*	**A specification** that provides a unique, persistent, (ideally) bi-directional, machine-actionable link between metadata and the data they describe.
**F4**	*Registry*	A **service** that indexes metadata and data and provides search over that index.
**A1.1**	*Communication Protocol*	A **specification** of how messages are structured and exchanged.
**A1.2**	*Authentication & Authorisation Service*	A **service** that mediates access to digital objects according to specifed conditions.
**A2**	*Metadata Preservation Policy*	A data **policy** that describes the conditions under which metadata should be provided in the future.
**I1**	*Knowledge Representation Language*	A language **specification** that enables knowledge to be processed by machines.
**I2**	*Structured Vocabularies*	A **specification** for a controlled list of uniquely identified and unambigous concepts with their definitions represented using web standards.
**I3**	*Semantic Model*	A **specification** that defines qualified relations between entities describing data or other digital objects according to the Linked Data principles. This can include semantic data models and ontologies.
**R1.1**	*Data Usage License*	A data **policy** that specifies legal restrictions on the reuse of the data.
**R1.2**	*Provenance Model*	A **specification** that specifies metadata describing the origin and lineage of data or other digital objects.
**R1.3**	*The FAIR Implementation Profile*	A **FAIR Implementation Profile (FIP)** is a list of declared technology choices intended to implement each of the FAIR Guiding Principles, made as a collective decision by the members of a particular community of practice.

The FER type version 2.0.0 was created in December 2023 by Barbara Magagna and Erik Schultes; GO FAIR Foundation Creative Commons Attribution Share Alike 4.0 International

**Table 2:** Data domain coverage for each AOP tool.

AOP tool	AOP	KE	KER	KEC	Stressor	Unique Gene ID	Pairwise Gene interaction	Species/taxa	Chemical Source	Chemical-gene association	Chem structure (ChemSpider, ChEMBL)	DTXID	Assay count	Pathway	Disease	Meta bolomic	Drug	GOterms	AOP-SNP associations	AOP-Haplotypes	Human Super-Pop	Linear AOP (LAOPs)	Users with Author Priv
AOP-DB	316	715			654	24609215	1	26554	1	1206437	0	654	406	110889	24166	0	1	26739	5217	2504	5	0	0
AOP Wiki	507	1822	2901		830	AD	0	AD	AD	0	0	0	0	0	0	0	0	0	0	0	0	0	335
Wik kaptis	379		1237			0	0	0	0	0	0	0	0	0	0	0	0	0	0	0	0	134651	0
Biovista vizit	470	1432			410	168362	0	7857	1	0	0	0	0	11810	62419	0	0	0	0	0	0	0	0
AOP-Wiki RDF	507	1490	2100		717	12662	0	167	1	0	1	403	0	0	0	1	1	0	0	0	0	0	0
AOP-WKI EXPLORER	316	715			654	0	0	AR	0	0		0	0	0	AR	0	0	0	0	0	0	0	0
AOPNetworkFinder	507	1490	2100		717	12662	0	167	1	0	1	403	0	0	0	1	0	0	0	0	0	0	0

1 of 0: presence absence or Do many counts to represent

Author dependant (AD)

Automated Extrac AR

**Table 3:** FAIR evaluation criteria to access interoperability and accessibility for AOP FERs (FSRs) (“A” and “I” part of FAIR principles).

Tools/resource	Open-source	Availability	Persistent-identifier provider(Miniting)	Integrate-ontologies	Capture provenance	Automated Parsing (unstructured text)	Linking with chemical Assay/reporting standards	Machine-readable format
AOP-Wiki		✕	✕	✕	✕		✕	✕
AOP-KB	✕	✕			✕			✕
AOP-Viki RDF	✕	✕		✕		✕	✕	✕
AOP-DB RDF	✕	✕		✕			✕	✕
AOP-DB	✕	✕		✕			✕	
CompToxAI	✕			✕			✕	
AOP-WIKI EXPLORER	✕			✕		✕		
AOP-network Finder	✕	✕						

## Data Availability

No data was used for the research described in the article.

## Box 1. FAIR concepts

### FAIR

A set of 15 principles intended to provide guidance on how to improve the Findability. Accessibility, Interoperability, and Reusability (FAIR) of digital assets. The principles emphasize the importance of both machine-actionability and human readability. The FAIR principles can be partitioned in two classes: those that address the *Information Technology*, i.e. the technical principles, and those that address the *Data Content*, i.e. the content-related, domain-relevant principles. * †

### Technical FAIR principles

The technical principles focus on the technical capabilities of the data infrastructure and are mostly agnostic to the content of the data. Their implementation supports general machine-actionability leading to interoperability across the Internet and is handled mainly by data system engineers. † †

### Content-related, domain-relevant FAIR principles

The content-related, domain-relevant principles focus on the implementation choices that must be made by practicing domain experts and include decisions regarding standards, minimum information requirements and (meta)data formats required to support human readability. †

### FAIR Enabling Resource (FER)

A FER is “any digital object that provides a function needed to achieve some aspect of “FAIRness” and is explicitly linked to one or more FAIR principle(s)” For example, an ontology can be a FER if it is formal, accessible, shared and broadly applicable for knowledge representation. Other examples include persistent identifiers. (meta)data templates, and standardized communication protocols, † † Further, FAIR Enabling Resources (FERs) can refer to the tools, infrastructures, services, and frameworks that support the implementation of the FAIR principles in data management and stewardship.

### FAIR Implementation Profiles (FIPs)

A FIP is generated digitally and describes the comprehensive set of implementation choices, i.e. FERs, made by a specific individual community of practice. A FIP can describe the FAlRness of a specific domain on a generic level or a specific data set. database or repository on a more detailed level. † †

### FAIR Convergence

A collection of community-specific FIPs compose an online resource referred to as a FIP Convergence Matrix which can be used to track the evolving landscape of FAIR implementations and inform optimization around reuse and interoperation. Overall and eventually. FIPs created by trusted communities will find widespread reuse among other communities, supporting accelerated and harmonized FAIR implementation within and particularly between domains. † †

*Adapted from WIKInson [.2016]; † Adapted from Schultes [. 2020]; † † Adapted from Schultes [. 2023].

## Abbreviations:

AI: Artificial intelligence
AOP: Adverse Outcome Pathway
AOP-DB: The EPA Adverse Outcome Pathway Database
AOP-KB: Adverse Outcome Pathway Knowledge Base
AOP-Tool: Third party tool that implements AOP data directly or indirectly from the AOP-Wiki XML
AOP-Wiki: The Adverse Outcome Pathway Wiki (https://aopwiki.org/)
DTSXID: A unique identifier assigned to a chemical substance within the US EPA’s Distributed Structure-Searchable Toxicity (DSSTox) database
e.AOP.Portal: Search engine for AOPs developed in the AOP-Wiki and Effectopedia modules of the AOP-Knowledge Base (note: AOP-KB Coordinating Group, in conjunction with OECD, has been working to phase out the e.AOP.Portal.)
EC: Executive Committee
ECCC: European Cybersecurity Competence Centre
EFSA: European Food Safety Authority
EHLC: National Institute of Environmental Health Sciences and US Environmental Protection Agency (NIEHS-US EPA) Environmental Health Language Collaborative
EPA: US Environmental Protection Agency
ESCA: Emerging Science in Chemicals Assessment (previously Extended Advisory Group on Molecular Screening and Toxicogenomics (EAGMST))
EU: European Union
FAIR: Findable, Accessible, Interoperable, Reusable
FC: FAIR contributing
FE: FAIR enabling
FER: FAIR Enabling Resource
FS: Fair supporting
GO FAIR: GO FAIR is a bottom-up, stakeholder-driven and self-governed initiative that aims to implement the FAIR data principles, making data Findable, Accessible, Interoperable and Reusable (FAIR)
IATA: Integrated Testing and Assessment is a hazard identification process that focusing on relevant biological pathways, using a combination of in silico, in vitro, and in vivo methods often referred to as NAMs
KE: Key event
KEC: Key Event Component Originally defined as “Event Component” in [11]
KER: Key Event Relationship
MIE: Molecular initiating event
NAMs: New Approach Methods
NIH: National Institutes of Health
NIEHS: National Institute of Environmental Health Sciences
NLP: Natural language processing (NLP)
OECD: Organization for Economic Co-operation and Development
PARC: Partnership for Assessment of Risk from Chemicals
SNP: Single Nucleotide Polymorphism
ToxCast: US EPA Toxicity Forecaster Program
US: United States
WP7: Work Package 7 (Partnership for the Assessment of Risks from Chemicals, PARC)
WPHA: Working Party on Hazard Assessment (OECD)
WNT: Working Party of the National Coordinators of the Test Guidelines Programme (OECD)
TF-IDF: Term frequency-inverse document frequency
CNNs: Convolutional Neural Networks
BiLSTMs: Bidirectional LSTM (Long Short-Term Memory) is an NLP technique used in text processing
